# Gynecology

**Published:** 1876-03

**Authors:** 


### III. Gynecology.

1. *Ovulation without Menstruation.* St. Germain. (*L'Union Médic. de Canada*, Dec., 1875.)

A woman married at 20, became pregnant in the early months of her married life, a few days after menstruation. She has since had eleven children, the last in her 45th year, having never menstruated throughout the entire period of 25 years.

She is a poor, hard-working woman, nervo-bilious temperament, small, but well-made, hips and shoulders broad, chest well developed, bright-eyed, and has rarely desired intercourse. She is now in her 78th year, and quite well.

2. *Some Points in Ovariotomy.* Bennett. (*Med. Record*, Nov., 1875.)

The writer considers two points: 1st. Position. The patient is placed on the edge of a table, and turned over almost upon her face, supported by two assistants. The sacs are then punctured and emptied as they present themselves, aided by the force of gravity. 2d. Treatment of pedicle. The author prefers the silk ligature, left depending at the bottom of the external incision. Five out of six patients thus treated, recovered—one case terminated fatally, from adherent and immovable sac. The clamp is objected to, because the pedicle is often short; tympanitis produces painful tension; vomiting and coughing produce a violent jerking, and may tear out the clamp, or induce inflammatory complications. It may also prevent cicatrization of the wound. The author's last operation was for removal of a multilocular tumor, weighing sixty or seventy pounds. The pedicle was tied in three parts, and in ten days the ligatures came away. In four weeks the patient was busied in her kitchen.

The writer considers the ligature the method of all others for securing the pedicle, which should be tied in two or three parts, the ligature not large, and drawn very tightly.

3. *Ovarian Disease, with Cases.* Bailey. (*Detroit Review of Med.*, January, 1876.)

The author reports a fatal case of ovariotomy from peritonitis. The tumor was multilocular, irregular, and flattened, as large as a placenta, filled with gelatinous substance of the consistence of the white of an egg. Upon this enlargement were flattened scales of calcareous matter. The pedicle was broad, the morbid formation adherent; and no attempt at removal was made.

*Post mortem:* Pus, tubercles and extensive adhesions were found.

In a second case, "something gave way" in the neighborhood of a large, well-defined tumor, in the left ovarian region, which at once seemed to become loose, rolling from side to side as she turned in bed. The abdominal cavity began to fill with a liquid. Death rapidly supervened.

4. *Pelvic Hæmatocele.* MORRILL. (*Bost. Med. and Surg. Jour.*, December, 1875.)

A woman, 24 years old, who had had one child and no miscarriages, suffered from slight leucorrhœa, and finally had sudden cessation of catamenia, followed by intense abdominal pain. Anodynes gave but partial relief, with some return of the menstrual discharge, when collapse occurred, with weak, intermittent pulse, sighing respiration, cold extremities, and a death-like pallor. Iron, ergot and tampons were used, and finally, uterine dilatation with tents, so that the finger could explore the fundus. No positive results.

Thirty-three days after the unmistakable signs of internal hæmorrhage, a rounded tumor, as large as an apple, appeared in the left iliac region, solid and dull on percussion. An elastic, egg-shaped swelling, its long diameter lateral, was detected behind the cervix. The sound, entered four and one-half inches, could be felt by the hand on the tumor, when the handle was depressed.

The tumor finally occupied the vagina. After exploring with the needle, a pint of bloody serum with clots was evacuated through an incision. Pus appeared later, from the wound, which was kept open by dilatation, and carbolic acid and permanganate of potash solutions were used as injections. The cure was rapid.

5. *Pelvic Hæmatocele and Pelvic Cellulitis.* BARKER, FORDYCE. (*Rich. and Louisville Med. Jour.*, January, 1876.)

Both occur in the pelvic cavity, immediately in contact with the uterus; both develop a tumor; both excite moderate pelvic peritonitis resulting in adhesions; both originate in predisposing disturbance of uterus or appendages.

Hæmatocele is associated with catamenial disturbance and abortion, never parturition. Cellulitis is frequently a disease of the puerperal period, or may follow abortion or inflammation of pelvic viscera. Hæmatocele frequently is ushered in by, or attends, uterine hæmorrhage; cellulitis rarely.

In hæmatocele, the tumor forms rapidly (in a few hours); for cellulitis, days are required.

In the former, the tumor is at first yielding and elastic, and indurates slowly with age; in the latter it is hard at first, and slowly softens. In the former, the tumor begins the disease; in the latter, it results from antecedent inflammation. In the former, the tumor is distinct from the uterus, of considerable volume, and displaces the womb laterally and anteriorly, the os backward; in the latter, the tumor belongs to the uterus, on one side or the other, and does not markedly displace it.

Finally, hæmatocele is ushered in by symptoms of nervous prostration and shock, and followed by those which attend pelvic and general peritonitis. Cellulitis begins with the general and local symptoms of inflammation.

6. *Peri-Uterine Hæmatocele Consecutive to Abortion.* SCHRANK. (*Medic. Chir. Centralblatt*, July, 1875.)

A woman, three months pregnant, lost blood after falling down stairs. Her physician discovered vacuity of the uterus, and the os dilated sufficiently to admit the extremity of the finger. Iced injections, ergot, and cold compresses upon the abdominal surface, were employed. Clots and débris of membrane were then expelled.

Two days after, the patient became quite pale, and fainted on making the slightest movement. Numerous clots were found in the vagina; and an abdominal tumor was discovered, voluminous, but neither painful nor tender. This was evidently unconnected with the uterus.

The escape of blood into the abdominal cavity was thought to be due to disease of the Fallopian tubes, and Dr. Schrank thinks that only in such cases, apart from, or in connection with, ovarian disease, is it possible for uterine secretions or injected fluids to traverse the canal.

7. *Peri-Uterine Adeno-Lymphangitis.* GUÉRIN. (*La France Médicale*, January 1, 1876.)

The author describes fully an exceedingly interesting case of peri-uterine inflammation of glands, lighted up by a septicæmic condition, probably resulting from induced abortion. Its features were those of a phlegmon of the large ligament. Metastatic abscesses occurred in the lungs, and pus was discovered in the pelvic veins.

8. *Rupture of Uterine Cyst, with Death from Hæmorrhage.* ANGEL. (*Charlestown Med. and Surg. Jour.*, January, 1876.)

A colored man, in attempting to effect intercourse with his wife, was resisted, and, in making forcible efforts, placed his knee upon her abdomen. She at once placed her hand upon her stomach, and complained of acute pain. Prostration, collapse and death occurred before midnight, without the attendance of a physician.

*Post mortem:* The woman seemed to be about 35 years old. There were about three quarts of clotted blood in the lower part of the abdominal cavity, containing a fœtus in the third or fourth month, completely surrounded by membranes. The pregnancy had evidently been intra-mural at the left utero-fallopian juncture. The uterine cavity was empty, and did not communicate with the external rent, a muscular partition inter vening. A well-marked cyst existed at this point, which did not communicate with the uterine cavity. Its external wall was thinned.

9. *Sarcoma Uteri.* SIMPSON, A. R. (*Ed. Med. Jour.*, January, 1876.)

The author gives details of four interesting cases. From these it appears that hæmorrhage, leucorrhœal discharge, destruction of the reproductive function, disturbance of function of neighboring organs, general cachexia, and no marked degree of pain, are the usual symptoms. Nulliparity does not seem to favor sarcomatous development.

The uterine enlargement is moderate, varying according to the size of the morbid mass, rarely so large as to form an abdominal tumor, unless there is a myo-sarcoma, or fibroid undergoing sarcomatous degeneration. When growing from the cervix or inverted uterus, a polypoidal body occupies the vagina. The enlarged uterus is freely movable, when there is not pelvic impaction or inflammatory adhesions. These are rare. The sound detects an increase in the size of the cavity, and a foreign body which readily bleeds. Tents are needful for exact diagnosis.

"My own case confirms the general verdict that we have here to do with a form of disease which, though it is not the cause of such intense suffering nor of such rapid constitutional deterioration, nor of such speedy death, as cancer, yet has a vicious tendency to recur after apparent complete removal, and to lead, sooner or later, to a fatal issue." In early and polypoidal cases, removal, repeated if necessary, is recommended, with the écraseur or galvano-caustic wire. Sometimes from brittleness, it is brought away piecemeal, with the curette, fenestrated like Récamier's, or cup-shaped like Simon's. In any case, it is desirable to lessen the immediate hæmorrhage by free application of perchloride of iron and hypodermic injection of ergotine. The profuse and sometimes fœtid discharge can be controlled by astringent and disinfectant injections; and the patient's general health will be kept up by a generous diet and the administration of tonic remedies.

10. *Tolerance of Sedatives in Malignant Uterine Disease.* DAY. (*Obstet. Jour. of Great Britain and Ireland*, January, 1876.)

(A.) A woman fifty-five years old, never pregnant, and having enjoyed good health up to the present illness, suffered from left inguinal pain for one year, (preventing sleep,) and a variable amount of uterine hæmorrhage. Menstruation had ceased two years before, but recurred and had never entirely disappeared since. The uterus was freely movable, and the os small. Immediately above the neck, which was well defined, the body dilated abruptly and involved the fundus. No enlargement was felt above the pubes. The swelling, which was rather hard and cartilaginous, occupied the upper and left side of the vagina. There was no ulceration nor vaginitis. There was much depression, weak pulse, face thin and haggard. A pessary, containing one-eighth of a grain of atropine, was ordered to be introduced into the vagina nightly. Glycerine on cotton wool was applied to the os in the day-time to relieve congestion. Bromide of potassium and quinine were given internally, and she was advised to remain in bed for a few days.

From the fifteenth of June, 1873, to the 10th of March, 1874, the date of her death, this patient suffered from intense, recurring pain. Fourteen hypodermic injections, each averaging three grains of the acetate of morphia, with one-third of a grain of atropine, were given from October 25 to December 30, 1874. From January 1 to January 22, 1875, two, and sometimes three injections were used daily, of the same strength. From January 22 to March 10, two and sometimes three injections were also daily used, each averaging five grains of morphia and half a grain of atropine.

These large injections never caused vomiting nor active cerebral disturbance. Full narcotism without danger to life was induced by the largest doses, no irritation following the punctures.

The writer cites one case where the introduction of one-sixth of a grain of the acetate of morphia under the skin of a lady forty years of age, induced sickness, faintness, disturbance of vision, and general tremor. It is important to combine atropine with the morphia, and to inject slowly and horizontally, avoiding veins.

(B.) In the second case, fully reported, one drachm of a solution of bimeconate of morphia failed to relieve, but half this dose, with 30 grains of chloral hydrate, never failed to induce sleep.

The author's conclusions are the result of a very careful and discriminating consideration of the subject of pain, as produced by malignant uterine disease in women of different constitutions and temperaments.

11. *Unusual Terminations of Uterine Fibroids.* PARVIN. (*American Practitioner*, November, 1875.)

Two cases are cited. In the first, a woman, thirty-eight years of age, childless after twenty years of married life, had ergot injected hypodermically for uterine fibroids, from which she had suffered four years. After its discontinuance, metrorrhagia returned ; the vagina and os were tamponed, the cervix divided, sponge tents were employed, and intra-uterine astringents applied. Finally, a stream of blood followed the withdrawal of two tents. An injection of one part of the muriated tincture of iron, with ten of warm water, was used freely to wash out the uterus, whose upper margin was slightly above a horizontal line at the umbilicus. The hæmorrhage was arrested, but temporary collapse was followed by death in forty-eight hours, from exhaustion.

In the second case, a woman thirty years old, had been married twelve years with two children, and one miscarriage. Leucorrhœa with profuse and irregular menses occurred four years previously, coincidently with "a lump" in the hypogastrium. There was emaciation, uterine pain. and hypogastric tenderness. A submucous uterine fibroid was established in the anterior wall, the uterine cavity measuring four inches and a half. While improving under general hygienic treatment, a purulent and shreddy discharge occurred, in which fibrous tissue was detected by the microscope. There has been great improvement in all respects since.

12. *Unusual Uterine Hæmorrhages.* BARKER, FORDYCE. (*N. Y. Med. Record*, January 29, 1876.)

The Professor, in some interesting remarks before the N. Y. Academy of Medicine, sums up the unusual causes of menorrhagia and metrorrhagia as follows : hyperæmia of the uterus due to perimetric exudation (exudative adhesions binding down the womb); acute ovaritis ; ovarian dysmenorrhœa ; acute ovarian displacements (prolapse from effort, shock, constipation) ; conditions of organs remote from uterus (obstructed portal, cardiac, renal circulation) ; disturbed cerebration from emotional causes ; malarial toxæmia ; exanthematous disease ; rapidly developed obesity (uterine plethora) ; chlorotic menorrhagia ; climacteric menorrhagia.

In the last named forms, B. uses, three times daily, rectal suppositories of cacao butter, and Squibbs' aqueous extract of ergot, for a week prior to menstruation. If the latter is prolonged, he introduces well into the cavity of the uterus, iodoform cylinders, made thus : R. Iodoform, dr. ijss. ; Gum. Tragacanth, grs. xv ; Mucil., q. s. M. Make ten cylinders, each 1¼ inches long.

13. *Pedunculated Uterine Fibroid Tumor—Pregnancy—Peritonitis—Death.* RODDICK. (*Canada Med. and Surg. Journal,* December, 1875.)

A healthy-looking woman, aged 29 years, married seven years without occurrence of pregnancy ; ceased menstruating for four months, and had mammary pain with morning sickness. She consulted her physician for an excruciating pain in the right inguinal region, which had for four months seized her without warning, compelling her at once to assume a sitting or recumbent position, and passing off in from three to five minutes, leaving her weak and prostrated. Recently, also, she had noticed a lump in her right groin. There had been also some constipation and enuresis, but no other outward syptoms.

On examination, the uterus was found to be enlarged, but not to the extent of the fourth month of pregnancy. A firm, movable mass of the size of a large hen's egg, longer in its vertical than in its horizontal diameter, was discovered somewhat higher than the right ovary. Pressure elicited considerable lancinating pain. A vaginal examination was refused.

In six days, intense nocturnal pain and incessant vomiting occurred. The features were pinched and anxious ; pulse, 112 and wiry ; tongue, furred ; general abdominal tenderness and pain in the right groin. The tumor was unchanged. Vaginal examination revealed the presence of a fœtus in utero, without digital perception of the tumor, which, however, participated in the forced movements of the womb.

Morphia, calomel and bismuth, with turpentine and poultices externally, produced relief for twelve hours, but the symptoms then returned. A blister followed by poultices was applied to the right side, and a solution of hydrocyanic acid, morphia and liquid bismuth, was substituted for the powders. The pulse was 120, small but regular, on the next day ; temperature, 101.5°. A simple enema was given for the constipation of three days. The vomiting became less constant, and the pain less severe ; but the general condition seemed more unfavorable. A considerable amount of blood was withdrawn by the application of a dozen leeches to the abdomen, when the latter was covered with a poultice. The pulse steadily rose to 130, the temperature to 102¼, and the respirations became hurried. Some iced champagne was given for relief of the vomiting.

The next day the patient had stercoraceous vomiting, with a small, irregular pulse of 140, subicteroid hue of skin, tongue deeply covered with yellow fur, pinched features and slight delirium. Death occurred on the ensuing evening, the fœtus with its investitures being expelled during the death struggle.

There was found at the autopsy, universal peritoneal injection, and partial intestinal congestion. The large, flaccid uterus had not contracted after the expulsion of its contents. Growing from the right side of the fundus, and attached to it by a peduncle about three-fourths of an inch in length, was a fibroid tumor of the size of a large hen's egg, the peritoneum in its vicinity being more injected than elsewhere—in places appearing almost gangrenous.

The following points of interest are discussed: 1. Could the fibroid growth, originally imbedded in the uterine wall, have prevented impregnation for nearly eight years? 2. Did it produce that peculiar catarrhal condition of the uterine mucous membrane so fatal to impregnation? 3. Are Barnes, Turner and others to be accepted, in teaching that pedunculated fibroid tumors are not to be regarded as serious complications of pregnancy?

14. *Inversion of Uterus, relieved by White's Method.* Coxe. (*N. Y. Med. Record*, December, 1875.)

A woman, 25 years old, married at 18, shortly after her confinement, was pale and in great pain, with a pulse of 180 and feeble. She soon rallied, but subsequently became much reduced, and was subject to frequent hæmorrhages. A tumor in the vagina, supposed to be a uterine fibroid, proved to be an inverted uterus.

White prepared her for operative procedure, with iron, quinine, generous diet, perfect rest, and ordered complete evacuation of the rectum. A ball of cotton cloth, saturated with horridly offensive putrid blood and discharge was, when withdrawn from the vagina, followed by the evacuation of a quantity of black, tarry fluid, of "infernal stench." This was a tampon a fortnight old, which had, for that time, bathed the uterus in a highly septic fluid. The vagina was at once cleansed.

By pushing up the tumor, White could feel plainly the inverted os through the relaxed and thin abdominal walls. Involution had not been interfered with.

The right hand, armed with a large rectal bougie, was passed into the vagina, the left making abdominal counter pressure. Partly by pressure, and partly by manipulation, the uterine walls soon began to yield—the patient being anæsthetized—and reduction was effected in six minutes. The "egg-beater" repositor was also used. The cure was complete.

15. *Uterine Asthma.* Waring, Curran. (*Practitioner*, December, 1875.)

Women with fibroid tumors of the uterus, or its appendages, appear to suffer more severely from asthma, than those who have any other form of tumor, the paroxysms being excessive and prolonged during the catamenial period. In three cases no evidence of the asthmatic complication appeared, until a day or two before menstruation occurred, and when the latter ceased, the asthma yielded without treatment. One lady, tapped five times for ovarian dropsy, is always thus affected when suffering from her periodic discharge, but the severity of the paroxysms is nothing compared to that occurring in patients with smaller and solid tumors. A woman, twice married without children, had a fibrous tumor of the uterus not much larger than an orange for fifteen years, without enlargement or pain, but at forty-five her periods became irregular, and asthma soon developed in an aggravated form. This case, as in the majority of similar instances, resisted the ordinary treatment of asthma.

In another patient, a uterine tumor occupied the right wall of the uterus for ten years; under the influence of iodine, it did not notably increase in size. After 40 years of age, however, it enlarged and produced asthma. The writer says he has seen patients blistered over the chest and covered with a profuse rash from some irritant, dosed with the ordinary remedies for asthma, and remain unrelieved. One lady informed him that she dreaded her menstrual epochs as she would a "fearful accident." The suffering of some is as severe as it can be described, inasmuch as the asthma is of the dry form, without expectoration to relieve it. The author has found little benefit from smoking, inhalations, or external applications, and believes the whole attention must be devoted to the uterus. He recommends, according to the period that has elapsed since the commencement of the catamenia, warm baths, ergot, bromide of potassium, belladonna, locally and internally, iron and strychnia in the intervals, with iodine locally. "New-fangled remedies" have failed in his hands. Subcutaneous injection is neither safe nor satisfactory. With prolapse, a tumor, and uterine asthma, a belladonna suppository is used. The ocular disturbance, occasionally complained of, is a mere temporary inconvenience. The belladonna extract may be spread upon lint and applied to the dorsal and lumbar spines.

(Asthma due to irritation reflected from the uterus does not differ from that occasioned by irritation elsewhere. Blisters and chest irritants are wretched applications in *any* such form, especially when occurring in a delicate and nervous woman. We cannot believe that this is the "ordinary treatment" in England. In America, aconite, and the iodide and bromide of potassium, have long supplanted other remedies.)

16. *Caustics in the Treatment of Diseases of the Cervix.* GILT. (*British Med. Jour.*, December, 1875.)

The author sums up the results of his observations as follows: 1st. In comparatively recent cases of endocervicitis, nitrate of silver, tincture of iodine or carbolic acid suffices. 2nd. Chronic cases of endocervicitis had best be treated by acid nitrate of mercury, or nitric acid. 3rd. Hyperchronic endocervicitis, with considerable cervical hypertrophy, requires potassa fusa cum calce, or some strong acid. He thinks Dr. Braithwaite is too general in his recommendation of nitrate of silver.

17. *Imperforate Os Uteri.* Dreyfous. (*Le Progrès Médical*, Oct. 16.)

A woman, 39 years of age, previously syphilitic, had suffered from no other prior disease, nor had ever been examined by the speculum—she had also never been cauterized nor used injections.

She had first menstruated at 20, and was well regulated for four years afterward; but then cessation of the catamenia had suddenly occurred, from plunging her hands into cold water. There were subsequent ill-defined periods, but, for twelve or thirteen years, there had been no vaginal discharge of blood nor supplementary menstruation. No leucorrhœa nor pregnancy had occurred.

On digital and speculum examination, no abnormal condition was discovered beyond the atresia, which was complete.

The interesting points in this case were: the excellent health enjoyed by a robust woman who had not menstruated for 13 years; the absence of every cause to explain the phenomenon, and the possibility of a congenital atresia; the early menstruation proceeded probably from the vaginal mucous membrane.

18. *Hæmatoma of the Vulva.* Canivet. (*Le Progrès Médical*, Dec., 1875.)

A woman, 26 years old, received, two years before, a kick with the foot upon the left labium majus. An ovid tumor soon occupied the entire labium, inferiorly projecting more than superiorly, and presenting a mucous and cutaneous face externally. It was as large as a hen's egg, indolent to the touch, and fluctuating; and seemed to be multilocular in character, so that the idea of puncture was abandoned.

Under anæsthetics, the tumor was readily enucleated above; but with greater difficulty below, in consequence of a prolongation which extended toward the ischium. In it were found numerous pockets, containing about 50 grammes of a dark, chocolate-colored liquid—altered blood. Bartholins' gland was intact; the seat of the hæmatoma was periglandular.

Sanguineous cysts, due to traumatic exudation, are rare. Huguier has seen but one case, and thinks that cysts filled with blood eventually become serous.

19. *Evil effects of Vaginal and Uterine Injections.* Padlock. (*St. Louis Med. and Surg. Jour.*, December, 1875.)

The author gives an excellent summary of the discussion in the Obstetrical Society of Dublin, on Feb. 13, 1875, elicited by Thomas More Madden's paper on "Metro-Peritonitis, following the Use of the Ordinary Female Syringe." Inefficiency and danger of the syringe are pointed out, not only in the ordinary application of medicaments to the vaginal and uterine surfaces, but in those employed after delivery. The central and peripheral orifices in the nozzle of the syringe, are alike liable to induce dangerous results. It is pointed out that the question whether there is transmission of fluids through the fallopian tube, is unessential, and subordinate to the question whether uterine colic, metro-peritonitis and peritonitis, supervene in consequence of either such actual transmission, reflected pain, neuralgia, or inflammation induced by neuralgia (Curling on the Testis), absorption, assimilation by chemical or vital processes, washing upward of morbid secretions from the uterus or cervix, etc.

It was shown that occasionally disastrous results follow in consequence of neglecting to exhaust the syringe of air, previous to its introduction into the vagina.

(Dr. Mac Arthur, of Chicago, was recently summoned to a lady, newly married, who was suffering from intense uterine pain and great nervous anxiety. He succeeded in removing from the cervix, the nozzle of a glass syringe, which had been broken off and left in the mouth of the uterus.)

20. *Irritability of Female Bladder for fifteen years, cured by Dilatation of Urethra and Neck of Bladder.* HEWETSON. (*Lancet*, December, 1875.)

An unmarried woman, 36 years old, suffered intensely from retention of urine, relieved by the catheter; the quantity of urine voided being such as must have distended the bladder nearly if not quite up to the umbilicus. She had been a schoolmistress, when, fifteen years before, she had had "inflammation of the bladder," terminating in a small abscess in the region of the urethra, which had spontaneously opened. Since then there had been headache, anorexia, "bearing down," depression of spirits, and nights disturbed every half hour or hour, by the necessity of passing small quantities of urine.

The orifice of the urethra was surrounded completely by warty growths of considerable size, and there was a tight sphincter ani. The rectum was baggy, and there was a small external pile. Uterus, catamenia and urine, normal.

The patient was anæsthetized, the warty growths removed, the pile snipped off, and the sphincter paralyzed, by stretching with the forefingers. During the next few weeks, there was relief of retention and pain in the motions of the bowels, but the vesical irritability remained, and in time the retention recurred.

On the 11th of April, the patient was again anæsthetized, and Weiss's female dilator was introduced into the urethra to the extent of about two inches, the blades being then *slowly* separated, and the urethra stretched so as to admit the forefingers within the bladder, while the parts were kept on the stretch. On closing the blades and withdrawing the instrument, the urethra contracted upon the little finger so as to sensibly grip it when introduced into the bladder, the coats of which were thickened. No foreign body was discovered there.

The irritability of the bladder and retention of urine were *completely* cured, without resulting incontinence.

21. *The Cotton Pessary.* PAGE. (*N. Y. Med. Jour.*, January, 1876.)

A piece of hard rubber rod, hollow or solid, as thick as a lead pencil and one inch and a half long, is bent with the fingers at any desired curve, after being passed through an alcohol flame. It is then laid upon a piece of cotton-batting, ten inches by eight, the long edge folded over about one inch and a half on each side. The rod is then placed at the short edge of the cotton, and firmly rolled the whole length of the piece, and then tightly wrapped with strong silk in the centre for one inch and a half, leaving a soft, compact and elastic ball at each end. (Small dumb-bell). Over the wrapping a piece of lint is sewed very smoothly, with the nap outside, and the pessary is complete. If the edges of the cotton are properly folded over, before the rolling, they will not fray or ravel, and the ends will be sufficiently protected.

